# American Medical Association

**Published:** 1878-04

**Authors:** 


### Editorial.

### American Medical Association,—Surgical Section.

It is but a few months before the American Medical Association meets in Buffalo, and it is with great pleasure that we print the following list of papers to be presented before the Surgical Section at the ensuing meeting.

The list kindly furnished us by the Secretary of the Section, E. T. Easley, M. D., of Little Rock, comprises the titles of articles received to date, and will be useful in allowing all to come prepared to discuss them understandingly. In our next issue we shall publish any additions that may be forwarded.

Address by Henry H. Smith, M. D., Chairman of the Section, "On Certain Points in the Pathology of the Bones, including Tubercles."

"On Disease Germs, their Nature, Origin and Relations, in cases of Wounds," by By B. A. Watson, M. D., Jersey City.

"On Septicæmia after Resections," by D. H. Weeks, M. D., Portland, Me.

"On Tracheotomy without Tubes," by Henry A. Martin, M. D., Boston, Mass.

"On Identity of Hospital Gangrene with Diphtheria," by Jno. T. Carpenter, M. D., Pottsville, Penn.

"On Permeability of Entire Alimentary Canal by Enemata with some Surgical Applications," by Robert Battey, M. D., Rome, Georgia.

"On Irritation of the Meta tarsal-Phalangeal Articulation in Valgus of the Great Toe," by Frank H. Hamilton, M. D., New York.

"On the Process of Repair in Wounds with and without Antiseptic Treatment," by Frederick Hyde, M. D., Cortland, N. Y.

"On Extirpation of the Thyroid Gland," by Julius F. Miner, M. D., Buffalo, N. Y.

"On Fractures at the Wrist," by Jno. H. Packard, M. D., Philadelphia, Pa.

"On Pathology and Treatment of Cancer," by Theodore A. McGraw, M. D., Detroit, Mich.

"On Perityphlitic Abscess," by D. M. Clay, M. D., of Shreeveport, La.

Owing to the distant residence of the Secretary, he requests that all papers to be presented in the session of the Section be forwarded at an early hour to Henry H. Smith, M. D,, Chairman of the Surgical Section, No. 1800 Spruce street, Philadelphia.

