# Correction

**Published:** 1878-06

**Authors:** 


Our attention is called to an error in the announcement of the title of Dr. FRANK H. HAMILTON'S paper, to be read before the A. M. A., in the April number of this *Journal;* it should read, "On Exsection of the Metatarso-Phalangal Articulation in Valgus of the Great Toe."

